# Artificial intelligence (AI) in psychological counseling: a double-edged sword demanding ethical precision

**DOI:** 10.17179/excli2025-8737

**Published:** 2025-11-05

**Authors:** Lysandro Pinto Borges, Athesh Kumaraswamy, Sasikumar Ponnusamy, Rajiv Gandhi Gopalsamy, Saju Madavanakadu Devassy

**Affiliations:** 1 Department of Pharmacy, Federal University of Sergipe (UFS), Aracaju, Sergipe, Brazil; 2 School of Sciences, Bharata Mata College (Autonomous), Thrikkakara, Kochi, Kerala, India; 3 Department of Medicine, University at Buffalo, Buffalo, New York, USA; 4 Division of Phytochemistry and Drug Design, Department of Biosciences, Rajagiri College of Social Sciences (Autonomous), Kochi, Kerala, India; 5 Postgraduate Program in Health Sciences (PPGCS), Federal University of Sergipe (UFS), Campus Prof. João Cardoso Nascimento, Aracaju, Sergipe, Brazil; 6 Department of Social Work & Rajagiri International Centre for Consortium Research in Social Care, Rajagiri College of Social Sciences (Autonomous), Kochi, Kerala, India; 7 Department of Social Work, Melbourne School of Health Sciences, University of Melbourne, Melbourne, Australia; 8 School of Social and Political Science, University of Edinburgh, Scotland, UK

## ⁯

In recent years, mental health has become a major global public health concern, with more people looking for help and treatment for a range of psychological problems and discomfort (WHO, 2022[11]). However, despite increased awareness and initiatives to lessen stigma, many people still face substantial obstacles when trying to access mental health services due to a lack of skilled professionals, financial limitations, and geographic barriers. To address these challenges, the use of technological innovations, especially artificial intelligence (AI)-driven tools such as chatbots, has become increasingly popular to deliver mental health interventions (Siddals et al., 2024[7]).

AI chatbots are interactive computer programs that simulate human communication using natural language processing and machine learning techniques to understand and respond to user inputs. They can analyze vast amounts of data and find patterns, providing doctors with important support in diagnosing mental illnesses and developing individualized treatment plans, while offering mental health seekers accessible tools for understanding and managing their conditions (Yadav, 2023[12]). AI-driven neurofeedback systems and brain-computer interfaces provide real-time feedback on brain activity, enabling individuals to develop self-regulation skills for emotional and cognitive control. AI-based machine learning (ML) approaches such as support vector machines (SVM), convolutional neural networks (CNN), and other deep learning models have demonstrated high accuracy in diagnosing cognitive disorders such as cerebral palsy, Alzheimer's, and seizure using magnetic resonance imaging (MRI) neuroimaging and electroencephalography data. Emotional AI uses data from facial expressions, voice, gestures, and physiological signals to identify emotional states, and improve human-device interaction. AI-powered therapeutic games and virtual reality environments offer immersive spaces for practicing emotional regulation. Additionally, AI tools can be used to analyze speech, eye movements, facial expressions, and social media content to detect early signs of disorders like mood shifts, depression, schizophrenia, and autism spectral disorders, supporting early diagnosis, personalized monitoring, and timely intervention (Thakkar et al., 2024[8]). AI tools also offer immediate and continuous psychological assistance and are available around-the-clock, making particularly valuable for individuals in crisis situations where prompt action is critical. Furthermore, AI has the potential to improve therapeutic approaches by suggesting coping strategies and customized treatment plans (Yadav, 2023[12]; Arjanto and Senduk, 2024[1]). Large language model (LLM)-based chatbots offer accessible, emotionally intelligent mental health support through user-friendly interfaces, providing non-judgmental dialogue, personalized feedback and guidance, and educational resources for self-awareness and care (Bassil, 2024[3]; Yoo et al., 2025[13]). Chatbots can evaluate stress levels, mood, sleep patterns, and user responses, recommend behavioral modifications, and advise users to seek medical care, including medication therapy (Han, 2025[5]).

While these AI advancements are promising, there remain a lot of ethical considerations around its use in mental health. One of the major considerations is the accuracy and reliability of these AI systems. As AI platforms are trained on pre-existing data, they may incorporate biases or contain inadequate information, resulting in incorrect diagnoses or poor treatment recommendations. Furthermore, AI tools may perpetuate existing structural inequalities or fail to account for specific cultural nuances that are relevant to mental health (Babu and Joseph, 2024[2]; Olawade et al., 2024[6]). Another significant issue that arises is the potential violation of people's privacy. Information misuse or data breaches could have serious repercussions for people, including making their mental health problems worse (Yadav, 2023[12]; Casu et al., 2024[4]). Another challenge is the ability of AI systems to offer genuine emotional support. Effective mental health treatment relies heavily on the therapists' ability to build rapport and trust, as well as on their empathic nature, qualities that even the most advanced AI may struggle to replicate (Arjanto and Senduk, 2024[1]). Without expert human oversight, unsupervised chatbots may engage in erratic interactions, disseminate false information or provide insufficient assistance, raising concerns about their ethical use and dependability as counseling tools (Bassil, 2024[3]). Furthermore, over-reliance on AI chatbots for emotional support may contribute to increased social isolation. In the absence of features for crisis intervention or appropriate governance mechanisms, users may be at risk during emergencies (Yoo et al., 2025[13]).

A thorough analysis of both the benefits and risks, along with the implementation of strict regulatory safeguards, is essential for ensuring the ethical and responsible use of AI in the field of mental health care. To address these risks, policy recommendations should include:

- National certification mandates for clinical trials of AI systems in mental healthcare prior to deployment
- Legal mandates for emergency situations, and the creation of a national registry of qualified mental health professionals ready for immediate help in emergency situations
- Stringent mental health-specific data protection laws
- Financial penalties for data breaches
- Mandatory inclusion of diverse demographic datasets during training
- The establishment of government-backed certifications to ensure AIs comply with safety and ethical standards
- Subsidized access to validated mental health AI tools for underprivileged communities,
- Ethical impact assessments as part of the regulatory approval process
- Regular audits of AI systems to verify adherence to ethical and safety standards with the findings published in publicly available reports
- The establishment of an independent regulatory body to monitor and handle issues related to AI misuse in mental healthcare
- The launch of national education initiatives partnering with schools, workplaces, and healthcare providers to inform the public about AI's uses and risks in mental heathcare (Thakkar et al., 2024[8]; van Kolfschooten and van Oirschot, 2025[10]).

Moreover, stakeholders should put feedback mechanisms in place, stay up to date with legal requirements, and work with mental health practitioners to develop and provide training in these tools in order to successfully integrate AI tools into mental health practice.

In conclusion, while AI technology offers significant potential benefits such as early detection, accessibility, nonjudgmental support, and cost-effectiveness, it is important to ensure that geographical disadvantages in respect of access to care are not reinforced in rural and remote areas. Moreover, this technology also raises concerns regarding its accuracy, privacy protection, ethical issues, and the potential to exacerbate social inequalities. AI's application in psychiatric counseling is therefore a double-edged sword that needs to be approached with equal parts of caution and hope. However, successfully balancing AI chatbot use with traditional mental health services can promote more inclusive and comprehensive care (Ueda et al., 2024[9]). AI may serve as an entry point into the mental health care system, but its outputs should be verified or supervised by qualified mental health professionals. 

## Notes

Rajiv Gandhi Gopalsamy and Saju Madavanakadu Devassy (Department of Social Work & Rajagiri International Centre for Consortium Research in Social Care, Rajagiri College of Social Sciences (Autonomous), Kochi 683104, Kerala, India; sajumadavan@gmail.com) contributed equally as corresponding author.

## Declaration

### Conflict of interest

The authors declare no conflicts of interest related to this work.

### Using artificial intelligence (AI)

The authors would like to disclose that QuillBot, an AI tool, was used to enhance the manuscript's language quality, readability, and vocabulary.

### Funding

No funding was received.
